# Social Media in Communicating Health Information: An Analysis of Facebook Groups Related to Hypertension

**DOI:** 10.5888/pcd12.140265

**Published:** 2015-01-29

**Authors:** Mohammad Al Mamun, Hamza M. Ibrahim, Tanvir Chowdhury Turin

**Affiliations:** Author Affiliations: Hamza M. Ibrahim, Department of Public Health, General Directorate of Health Affairs in Tabuk Region, Ministry of Health, Kingdom of Saudi Arabia; Tanvir Chowdhury Turin, Department of Family Medicine, University of Calgary, Alberta, Canada.

## Abstract

**Introduction:**

We studied Facebook groups related to hypertension to characterize their objectives, subject matter, member sizes, geographical boundaries, level of activity, and user-generated content.

**Methods:**

We performed a systematic search among open Facebook groups using the keywords “hypertension,” “high blood pressure,” “raised blood pressure,” and “blood pressure.” We extracted relevant data from each group’s content and developed a coding and categorizing scheme for the whole data set. Stepwise logistic regression was used to explore factors independently associated with each group’s level of activity.

**Results:**

We found 187 hypertension-related Facebook groups containing 8,966 members. The main objective of most (59.9%) Facebook groups was to create hypertension awareness, and 11.2% were created primarily to support patients and caregivers. Among the top-displayed, most recent posts (n = 164), 21.3% were focused on product or service promotion, whereas one-fifth of posts were related to hypertension-awareness information. Each Facebook group’s level of activity was independently associated with group size (adjusted odds ratio [AOR], 1.02; 95% confidence interval [CI], 1.01–1.03), presence of “likes” on the most recent wall post (AOR, 3.55, 95% CI, 1.41–8.92), and presence of attached files on the group wall (AOR, 5.01, 95% CI, 1.25–20.1).

**Conclusion:**

The primary objective of most of the hypertension-related Facebook groups observed in this study was awareness creation. Compared with the whole Facebook community, the total number of hypertension-related Facebook groups and their users was small and the groups were less active.

## Introduction

Because the Internet is a vast source of information, people are increasingly using it to search for health or medical information (1,2), to participate in support groups (2,3), and to consult with health professionals (2). Online support groups offer people complementary and supplementary care, create networks with peers to share common experiences, increase problem-solving skills, and increase confidence in making life-improving changes (2,4). Because Internet sites are convenient, easily accessible, available 24 hours a day, cost-effective, and informative (2), people search the Internet for information about medical conditions and treatment more frequently than they communicate with their physicians about their health care (5).

Social media are convenient means of communication by which people create, share, and exchange information and ideas across Internet-based communities and networks throughout the world (6). Social media sites are popular because users can easily generate content and instantaneously make that content widely available and accessible (7). In general, user-generated content refers to a wide range of information, photos, pictures, videos, tags, reviews, and play-lists created and posted by registered users belonging to a particular social media site (6). YouTube, Twitter, Facebook, MySpace, Google+, and Flickr are some well-known examples of popular social media (6). Facebook (www.facebook.com) is the most popular and largest social networking platforms and comprises more than one billion active users (8–10). A major feature of Facebook is a user’s “wall,” which is a space where either the users or visitors can post statuses or comments, upload videos or photos, or attach files. Another Facebook feature is the “like” button: readers can click “like” as a way to indicate their “liking” of or agreement with a comment, photo, video, or other post. 

Hypertension, or high blood pressure, is a global public health burden that contributes to morbidity, mortality, and health care cost in both developing and developed countries (11–13). A 2013 study reported that 67% of American adults living with hypertension were Internet users, and 58% of them accessed a website that provided information about a specific medical condition or problem (14). Since appropriate controlling of hypertension reduces the risk of adverse health events, support activities through social networking sites could be effective in hypertension control. Although numerous studies focused on people’s use of various social media sites in the context of diseases other than hypertension (3,5,15–19), studies on the use of social media sites for hypertension-related information and communication are limited.

Literature is limited on how Facebook groups are used by people with hypertension-related diseases or how these groups foster awareness regarding hypertension among Internet users throughout the world. We studied Facebook groups related to hypertension to characterize the following: a) objective (the main purpose for creating each group), b) subject matter or main discussion topic of each group (eg, type of hypertension), c) number of members in each group, d) geographic boundaries (are group activities targeted to a specific regional population or the global population), e) level of activity (frequency with which group members post to the site), and f) type of user-generated content posted on the wall of each group (ie, individual member’s health status, photos, videos, events, or files), their “likes,” and comments regarding the items posted.

## Methods

On September 14, 2013, we performed a systematic search of Facebook using the keywords hypertension, high blood pressure, raised blood pressure, and blood pressure. We limited our search to groups that were accessible to anyone having a Facebook account and used the options provided by Facebook’s built-in search engine. For example, while we typed “open groups” and “hypertension” consecutively in the Facebook search box, numerous automated texts (search options) appeared in the search box. Among those automated search options, we selected only “open groups named hypertension” to obtain a precise search result. Reviewing all search results, we excluded the groups that contained subject matter either unrelated to hypertension or not in English (Figure). Facebook pages for individual users, organizations, events, and applications were not included in our study. “Facebook groups” and “Facebook pages” are different aspects of the social network site: Facebook groups provide a closed space for small groups of people to share their common interests and to express their opinion, whereas Facebook pages allow real organizations, businesses, celebrities, and brands to communicate broadly with people who like them (20). For our study, we focused on Facebook groups and did not include Facebook pages.

**Figure:**
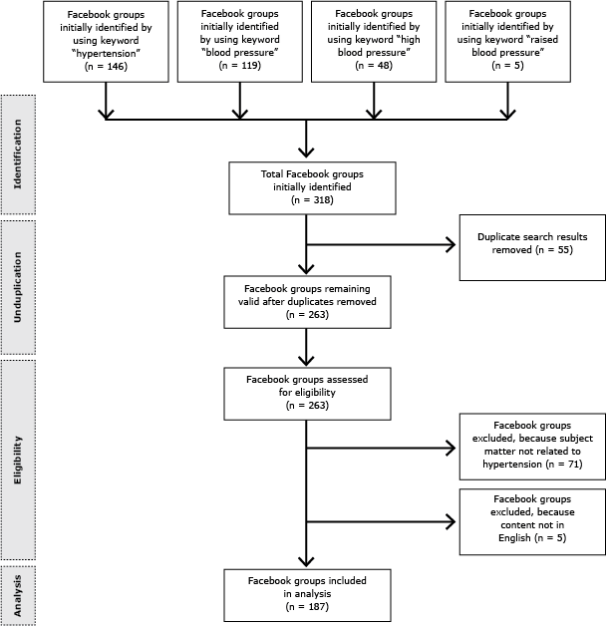
Process for including Facebook groups related to hypertension in analysis of Facebook groups related to hypertension.

Two researchers (M.A. and T.C.T.) extracted the following data from the content of each eligible group: title of the group; Web address (URL); introductory description; total number of members; most recent top-displayed wall post with posting date, number of “likes,” and comments on that post; and an indication as to the presence or absence of photos, videos, events, and attached files on the group’s wall. Using the method of content analysis (21–23), M.A. and T.C.T. developed a unified coding and categorizing scheme for the whole data set by reviewing the content of the first 100 groups on the basis of the theme present in each group. The main objective of each group was derived from the content of the group title, introductory description, or any message posted by the group creator or administrator. M.A. and T.C.T. independently assigned a specific code for each main objective by using a coding scheme we developed for this study. We assigned a specific code for each of the main objectives, which ultimately led to the identification of 7 major categories of hypertension groups: (a) awareness-creating groups; (b) support groups for patients and caregivers; (c) experience-sharing groups; (d) fundraising groups; (e) product promotion groups; (f) research-conducting groups; (g) health professionals groups. The same 2 coders also coded each of the top-displayed recent wall posts on the basis of the theme of that particular wall post. The intercoder reliability (agreement between two coders) was measured by calculating the percentage of agreement episodes between both coders during the coding process (22). Disagreements between the 2 coders were resolved by discussions until a consensus was reached, or by an adjudication by the third investigator (H.M.I.).

According to the date of the most recent wall post or comment, we placed the Facebook groups into 2 categories: active (most recent wall post or comment was posted on or after January 1, 2013, and before September 14, 2013, and less active (most recent wall post or comment was posted before January 1, 2013). Because we collected data for our study during September 2013, we arbitrarily chose a cutoff point of January 1, 2013 (the beginning date of the same year), to measure the recency of the latest wall post or comment of each Facebook group. Recency measure was preferred, because it was simpler and less time-consuming than other measures of activity such as counting total number of wall posts, comments, likes, or re-posts in each Facebook group; and measuring total number of past events in each group could give an impression of the volume of overall activities, but not necessarily the recency of those activities.

Descriptive statistics comprised the calculation of mean for continuous variables and absolute numbers and percentages for categorical variables. Categorical variables were summarized in 2 frequency tables showing corresponding absolute numbers and percentages. From our everyday experience of Facebook use, it is evident that many Facebook groups become inactive or less active as time goes on. From this perspective, we emphasized and measured the level of activity of the Facebook groups and selected level of activity as an outcome variable for logistic regression analysis.

Stepwise logistic regression (backward elimination method) was used to explore the factors independently associated with the level of activity of the Facebook groups related to hypertension. We preferred stepwise logistic regression because a) our data consisted of multiple potential explanatory variables, b) the important covariates on which to base the model-building were unknown, and c) the outcome we studied was relatively new. A stepwise selection procedure can screen numerous variables in a quick and effective way and can fit numerous logistic regression equations simultaneously (24).

All variables having a univariate level of significance *P* < .10 were selected for inclusion in the base model for multivariable analysis. Unadjusted and adjusted odds ratios (AORs) along with 95% confidence intervals (CIs) were calculated. All reported *P* values were 2-sided, and *P* < .05 was considered significant. Model fit was assessed by the Hosmer–Lemeshow test. All analyses were performed by using SPSS version 16.0 (IBM Corporation).

## Results

Our search results yielded 263 groups on Facebook; 187 groups were eligible for data extraction and analysis after exclusion criteria were applied (Figure). A total of 8,966 Facebook users were members of these eligible groups related to hypertension, and the membership size of each group ranged from 1 to 2,161 individuals. Overall intercoder reliability in our study was 89.8%.

Table 1 shows the general characteristics of the Facebook groups included in the study. Group activities were restricted to a particular geographical location (country or region) for 15% of the groups as indicated by the title of these groups (eg, Singapore Hypertension Club, Pulmonary Hypertension Support Group for Southern Alberta, High Blood Pressure North Florida ), and the remaining 85% of the groups were considered global. By analyzing the main objective of each group we identified the following 7 major categories of hypertension groups by their group descriptions: a) awareness-creating groups (“Our goal is to make people aware about hypertension”), b) support groups for patients and caregivers (“This is a support group for people living with pulmonary arterial hypertension and their family and friends”), c) disease experience-sharing groups (“I am 27 and I have 3 beautiful kids and I am now living with severe idiopathic pulmonary hypertension. I need to know how some of you deal with knowing you are fighting for your life every single day and not break down”), d) fundraising groups (“This group is for those that wish to help us by offering donations or by volunteering yourself, your time, your talents for the organization”), e) product-promotion groups (selling online-generic Lopressor (metoprolol) at discount prices), f) research groups (High Blood Pressure Clinical Research Trial], g) health professional groups (Libyan Academy of Hypertension). Our analyses revealed that most (59.9%) of the Facebook groups were created mainly to promote hypertension awareness. Table 1 lists the key objectives of the Facebook groups and the number and percentage of groups with each objective.

**Table 1:** Number and Percentage of Characteristics of Facebook Groups Related to Hypertension (N = 187), 2013

Characteristic	Number (%)
**Geographic **	
Global	159 (85%)
Regional	28 (15%)
**Subject focus**	
Hypertension	76 (40.6%)
Pulmonary hypertension	56 (29.9%)
Intracranial hypertension	27 (14.4%)
Hypertension plus other diseases^a^	28 (15%)
**Principal objective**	
Awareness creation	112 (59.9%)
Providing supports to patients and caregivers	21 (11.2%)
Sharing disease experiences and life stories	20 (10.7%)
Fund-raising for relevant organizations	14 (7.5%)
Product or service promotion	7 (3.7%)
Conducting studies or surveys	7 (3.7%)
Networking among health professionals	6 (3.2%)
**Level of activity**	
Active (latest activity on or after January 1, 2013)	52 (27.8%)
Less active (latest activity before January 1, 2013)	135 (72.2%)

a Other diseases include diabetes, heart diseases, kidney diseases, obesity, stress, and anxiety.

Most (21.3%) of the top-displayed most recent wall posts focused on promoting a product or service related to hypertension. The themes of the Facebook groups’ top-displayed most recent wall posts with respective proportions and percentages are illustrated in Table 2. Of the 187 groups analyzed, 27.8% had activity since January 1, 2013, and 72.2% groups had no activity since that date (Table 1). Stepwise logistic regression analyses showed that the level of activity of the hypertension-related Facebook groups was independently associated with group size (AOR, 1.02; 95% CI, 1.01–1.03), presence of at least 1 “like” on the most recent wall post (AOR, 3.55; 95% CI, 1.41–8.92), and presence of at least 1 attached file on the group wall (AOR, 5.01; 95% CI, 1.25–20.1). These associations should be interpreted with caution, because the variable “group-size” was not normally distributed and showed relatively small magnitude in CI, and the variable “presence of attached file” showed wide CI, possibly because of the small number of analyzed Facebook groups). Further studies with larger numbers may confirm our findings. Finally, the Hosmer–Lemeshow test indicated that the logistic regression model fitted the data in our study well (*P* = .24). Table 3 illustrates the association of selected characteristics with level of activity of the Facebook groups.

**Table 2:** Number and Percentage of Facebook Groups Related to Hypertension (N = 187) with User-Generated Content on Their Facebook Walls, 2013

User-Generated Content	Number (%)
**Wall post**	
Yes	164 (87.7%)
No	23 (12.3%)
**“Like” on top-displayed recent wall post (n = 164)**	
At least one “like”	40 (24.4%)
No “like”	124 (75.6%)
**Comment on top-displayed recent wall post (n = 164)**	
Yes	29 (17.7%)
No	135 (82.3%)
**Theme of top-displayed recent wall post (n = 164)**	
Product or service promotion	35 (21.3%)
Sharing hypertension awareness related information	33 (20.1%)
Sharing an external web address related to health	22 (13.4%)
Query to members for a particular information	16 (9.8%)
Greeting, wishing or thanking message	15 (9.1%)
Event promotion	14 (8.5%)
Description of group interest	13 (7.9%)
Sharing disease experience or life story	11 (6.7%)
Fund-raising message	5 (3%)
**Photo posted on group wall (n = 164)**	
Yes	84 (51.2%)
No	80 (48.8%)
**Video posted on group wall (n = 164)**	
Yes	9 (5.5%)
No	155 (94.5%)
**Event posted on group wall (n = 164)**	
Yes	2 (1.2%)
No	162 (98.8%)
**Attached file posted on group wall (n = 164)**	
Yes	14 (8.5%)
No	150 (91.5%)

**Table 3:** Association of Selected Characteristics With Level of Activity of Facebook Groups Related to Hypertension, 2013

Characteristic	Low Activity^a^ (n = 135)	High Activity^b^ (n = 52)	Unadjusted OR		Adjusted^c^ OR	
			OR (95% CI)	*P* Value	OR (95% CI)	*P* Value
**Geographic distribution, n (%)**						
Global	118 (74.2)	41 (25.8)	0.54 (0.23–1.24)	.15	—	—
Regional	17 (60.7)	11 (39.3)	1 [Reference]		—	—
**Subject focus, n (%)**						
Hypertension	62 (81.6)	14 (18.4)	1 [Reference]		—	—
Pulmonary hypertension	36 (64.3)	20 (35.7)	2.46 (1.11–5.46)	.03	—	—
Intracranial hypertension	19 (70.4)	8 (29.6)	1.86 (0.68–5.12)	.23	—	—
Hypertension plus other diseases^d^	18 (64.3)	10 (35.7)	2.46 (0.94–6.47)	.07	—	—
Group size, mean	6.8	154.6	1.03 (1.01–1.04)	< .001	1.02 (1.01–1.03)	.003
**Principal objective, n (%)**						
Awareness creation	81 (72.3)	31 (27.7)	0.38 (0.07–2)	.25	—	—
Providing support to patients and caregivers	15 (71.4)	6 (28.6)	0.4 (0.06–2.57)	.33	—	—
Sharing disease experiences and life stories	11 (55)	9 (45)	0.82 (0.13–5.08)	.83	—	—
Fund raising for relevant organizations	11 (78.6)	3 (21.4)	0.27 (0.03–2.11)	.21	—	—
Product or service promotion	7 (100)	0 (0)	< 0.001	.99	—	—
Conducting studies or surveys	7 (100)	0 (0)	< 0.001	.99	—	—
Networking among health professionals	3 (50)	3 (50)	1 [Reference]		—	—
**User generated content, n (%)**						
Presence of wall post	112 (68.3)	52 (31.7)	> 999.9	.99		
Presence of “like”^e^	15 (37.5)	25 (62.5)	5.99 (2.77–12.92)	< .001	3.55 (1.41–8.92)	.007
Presence of comment^e^	15 (51.7)	14 (48.3)	2.38 (1.05–5.4)	.04	—	—
Presence of photo	44 (52.4)	40 (47.6)	5.15 (2.44–10.89)	< .001	—	—
Presence of video	5 (55.6)	4 (44.4)	1.78 (0.46–6.93)	.4	—	—
Presence of event	0 (0)	2 (100)	> 999.9	.99		
Presence of attached file	4 (28.6)	10 (71.4)	6.43 (1.91–21.62)	.003	5.01 (1.25–20.1)	.02
**Theme of top-displayed recent wall post, n (%)**						
Product or service promotion	31 (88.6)	4 (11.4)	0.15 (0.03–0.75)	.02	—	—
Sharing hypertension-related information	18 (54.5)	15 (45.5)	1 (0.25–3.94)	1	—	—
Sharing an external web address related to health	15 (68.2)	7 (31.8)	0.56 (0.13–2.48)	.44	—	—
Query to members for a particular information	12 (75)	4 (25)	0.4 (0.08–2.06)	.27	—	—
Greeting, wishing or thanking message	10 (66.7)	5 (33.3)	0.6 (0.12–2.97)	.53	—	—
Event promotion	9 (64.3)	5 (35.7)	0.67 (0.13–3.35)	.62	—	—
Description of group interest	9 (69.2)	4 (30.8)	0.53 (0.1–2.84)	.46	—	—
Sharing disease experience or life story	6 (54.5)	5 (45.5)	1 [Reference]		—	—
Fund raising message	2 (40)	3 (60)	1.8 (0.21–15.41)	.59	—	—

Abbreviations: CI, confidence interval; —, not applicable; OR, odds ratio.

a Group’s most recent wall post or comment was posted on or after January 1, 2013

b Group’s most recent wall post or comment was posted before January 1, 2013

c Adjusted for subject focus, group size, presence of “like” on top-displayed recent post, presence of comment on top-displayed recent post, presence of photo, presence of attached file, and theme of the top-displayed recent wall post.

d Other diseases include diabetes, heart diseases, kidney diseases, obesity, stress, and anxiety.

e On top-displayed recent wall post.

## Discussion

In recent years, many people with diagnosed diseases have formed Internet communities and used the Internet as a platform for accessing timely and relevant health-related information (1,2). Since Facebook is one of the largest social network sites whose user base includes people of all ages and backgrounds, understanding how Facebook groups are raising health awareness among users can assist in developing interventions for health information, education, and communication. Although several studies explored the content and role of Facebook groups pertinent to different chronic diseases such as diabetes (5,15,16), breast cancer (3,15), and colorectal cancer (15) and risk factors such as tobacco use (25), our study is the first to analyze the content of the Facebook groups related to high blood pressure. Our study echoes the importance of community hypertension programs in the era of mobile technology and social media emphasized by Logan et al in a recent article (4).

Our study found approximately 9,000 Facebook users connected with hypertension-related groups. This number is a small contingent of Facebook’s more than 1 billion active-users and suggests that hypertension awareness has a small sounding board on the social network site. Our study revealed that activities of the hypertension-related Facebook groups were mostly (85%) global or international, which was similar to the finding of a case study of a diabetes group (16). Given that our search was limited to English and thus excluded Facebook groups that use other languages, our estimate of geographic boundaries of the groups (global or regional) may not reflect the real situation.

According to our study, most (59.9%) hypertension-related Facebook groups were formed mainly for hypertension-related awareness, whereas Bender et al reported that most (44.7%) of the breast cancer-related Facebook groups were created chiefly for fundraising purposes (3). Farmer et al found that only 28.1% of Facebook groups related to noncommunicable disease were providing support to patients and caregivers (17). Similarly, in our study we found a low proportion (11.2%) of hypertension-related groups working as support groups for patients and caregivers. It is not surprising that our study showed that awareness creation is the main objective of most hypertension-related Facebook groups, rather than fundraising or supportive care activities. Hypertension treatment is less expensive than treatment for many other chronic diseases. Regarding group members’ contributions to the groups, we found few user-generated contributions , which we assessed as cumulative “likes” and comments (24.4% and 17.7%, respectively), on the top-displayed recent wall post indicating that most group members were ”lurkers,” (a member of an online community who observes but does not actively take part] (26), rather than “posters.” This finding is not unusual for an Internet community (26,27) and is also consistent with the findings of the study on breast cancer-related Facebook groups conducted by Bender et al (3).

While analyzing the themes of the top-displayed most recent wall posts, we observed that 21.3% of wall posts were related to promotional products or services for people with hypertension, which suggests the active presence of representatives of medical or pharmaceutical companies among the group members. This finding is troubling, because of the unknown quality and safety of products and services promoted in open environments such as Facebook groups. Similar to our study’s result, Kumar et al reported that 20.1% of hypertension-based YouTube videos contained product advertisements, and many of them advocated unproven alternative treatments of hypertension, which raises concerns about patient safety (28). Moreover, the World Health Organization pointed out the potential harm to individuals’ and the public’s health from medical products sold via the Internet (29).

We found that about one-fifth of the top-displayed and most recent wall posts were focused on providing group users with information related to hypertension awareness. Two qualitative studies about diabetes-related Facebook groups conducted by Greene et al and Zhang et al reported that more than 60% of wall-post topics were based on sharing information about diabetes (5,16). However, neither of those studies (5,16) nor our study examined the scientific accuracy of the information posted on Facebook group walls. Although vetting the accuracy of the information posted to the hypertension-related Facebook groups’ walls was beyond the scope of our project, some studies have explored the accuracy of health-related information available on the Internet (28,30).

Our study has several limitations. We collected data only from the “open” Facebook groups, that is, groups that were completely accessible to the public. Unfortunately, we were unable to collect information from closed or secret groups because of their privacy settings. Thus, we do not know if closed and secret Facebook groups related to hypertension show similar or different content than open groups. This study reflects only the content of hypertension-related Facebook groups and does not represent data from other large social media sites such as Twitter, Myspace, or Google+. Moreover, our data collection was restricted to groups operated in English, although Facebook is available in many other languages. Only the top-displayed, most recent wall post from each Facebook group was assessed, and other wall-posts were not considered. Although we calculated intercoder reliability, intracoder reliability was not assessed. Membership in the 187 Facebook groups may have overlapped. Unfortunately, we were not able to determine how many of the groups were interconnected or to account for membership overlap. In addition, there is the possibility that some users visit the hypertension-related open Facebook groups but do not join them for privacy reasons. Furthermore, we were unable to determine the sex or race of Facebook group members.

In conclusion, this study was a systematic search of Facebook’s open hypertension-related groups. Because it is the first of its kind, our study sheds light on a small Internet community and opens the door to future research. We found that the number of English hypertension-related Facebook groups and their users was small compared with the whole Facebook community, and the groups’ activity levels were low. Despite their small number and low activity level, the hypertension-related Facebook groups provide a sounding board for those affected by the chronic illness, inasmuch as most hypertension-related Facebook groups boost awareness. Facebook groups may be a useful platform for creating hypertension awareness across a global population. 
